# Making sense of zebrafish neural development in the Minervois

**DOI:** 10.1186/1749-8104-2-15

**Published:** 2007-08-08

**Authors:** Alain Ghysen, Christine Dambly-Chaudière, David Raible

**Affiliations:** 1 Lab. Neurogenetics, INSERM U881, Université Montpellier 2, 34095 Montpellier, France; 2 Department of Biological Structure, University of Washington, Seattle, WA 98195-7420, USA

## Abstract

The meeting 'From sensory perception to motor output: genetic bases of behavior in the zebrafish embryo' was held at Minerve (South of France) on March 16–18, 2007. The meeting site was beautifully situated in the heart of the Minervois wine country, and its remoteness promoted conversations and interaction over the course of the program. The meeting covered neurogenesis and eye development on day 1, ear and lateral line development on day 2, and brain connectivity and behavior on day 3. Underlying all sessions, however, ran the growing importance of live imaging, an approach that takes full advantage of the transparency of fish embryos and early larvae, as illustrated by several movies and links in this report.

## Background

The meeting 'From sensory perception to motor output: genetic bases of behavior in the zebrafish embryo' [1] was held at Minerve (South of France) on March 16–18, 2007. The meeting site was beautifully situated in the heart of the Minervois wine country, and its remoteness promoted conversations and interaction over the course of the program [2].

The sessions were roughly organized in terms of neurogenesis and eye development on day 1, ear and lateral line development on day 2, and brain connectivity and behavior on day 3. Underlying all sessions, however, ran the growing importance of live imaging, an approach that takes full advantage of the transparency of fish embryos and early larvae. The imaging methods are expanding at a fast rate, with a rapidly increasing panoply of green fluorescent protein (GFP)-expressing reporter lines, fluorescent protein spectral variants, and photoactivatable compounds. Many fascinating movies were presented at the meeting exploring crucial steps in cell migration (R Köster, D Gilmour, H Okamoto or neural differentiation (A Sagasti, K Kwan, W Harris, J Schweitzer, and documenting behavioral responses (S Jesuthasan, T Burt de Perera, F Engert, M Granato. There is no doubt that this level of analysis will play a major role in future progress.

## Eye development

**Figure 1:**
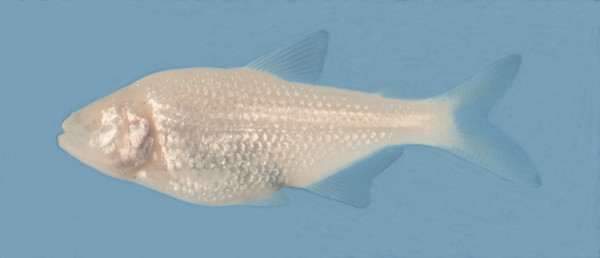
*Astyanax fasciatus*, cave form. Picture provided by B Jeffery.

Bill Jeffery introduced the session on vision with a talk on his pet fish, the Mexican tetra, *Astyanax fasciatus*. This species has two forms: a surface form provided with pigment and eyes, and a cave form that is unpigmented and blind (Figure 1). The eyes of the blind form appear normally but begin to degenerate as early as two days after fertilization. Blind forms appeared independently at several locations less than a million years ago and possibly as recent as 10^5 ^years ago for some of them, suggesting that eye loss occurred rapidly and may be under strong selection.

One common-sense explanation for the selective advantage of eye loss might be energy conservation. Jeffery showed, however, that the degenerate retina of the blind fish still has a proliferating rim (the ciliary marginal zone) similar to that of the eyed fish, but gets rid of the newly formed cohorts of retinal cells by continuous apoptosis. This observation makes the 'energy-saving' explanation somewhat unlikely, although not impossible. He added that it could be more energy efficient to kill newly born cells than to evolve new mechanisms to uncouple stem cell division in the retina and central nervous system. Because blind fish have an expanded mouth and increased number of taste buds, an alternative explanation for eye loss is indirect selection based on an advantageous increase in mouth development.

Jeffery found that eye loss is due to an expanded domain of expression of the gene *sonic hedgehog *(*shh*), an expansion that also leads to the observed changes in head and mouth morphology. He proposed that there is a trade-off between vision and food detection and that this sensory trade-off played a major role in driving eye degeneration. Other factors are probably at work, though, since only a subset of the ten or so genetic factors (quantitative trait loci (QTL)) implicated in the differences between eyed and surface forms affect both the eyes and the taste buds. Because the surface and cave forms are fully interfertile, QTL analysis can be applied at many levels, for example, development, morphology, and behavior. *Astyanax *may well turn out to be an excellent system to study evolution caught in the act.

**Figure 2:**
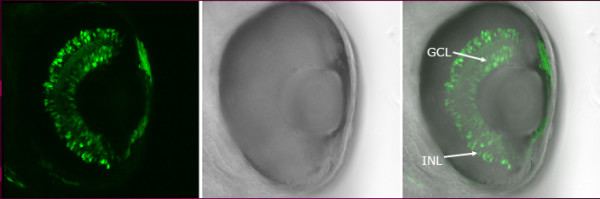
Example of a *shh *enhancer specific for the ganglion cell layer (GCL) and inner nuclear layer (INL) of the retina, driving expression of GFP (72 hpf embryo carrying the -2.shh:gfpAC11 transgene). Figure provided by Saradavey Rathnam in U Strähle's lab.

As a logical continuation of Jeffery's talk, Uwe Strähle discussed the regulation of *shh *expression in eye development (Figure 2). Fgf signals are prime candidates for regulating *hedgehog *expression in the eye. The Ets type transcription factors Erm and Pea3 act as a transcriptional readout of Fgf signals, and are expressed in the eye at appropriate times to regulate *shh*. These factors specifically bind to *shh *eye enhancers. Using morpholino antisense oligonucleotides against both factors effectively blocked expression from the eye-specific promoter.

Given the very high conservation of *shh *expression patterns between fish and mouse, and the presence of highly conserved stretches of non-coding DNA upstream and in introns of the *shh *coding region, one might have expected the function of *shh *enhancer regions to be well conserved. This, however, turned out not to be the case: nearly identical expression patterns turn out to be driven by totally different enhancers in fish and mouse. In a sense, this ambiguous situation is reminiscent of the so called 'phylotypic stage', which appears highly similar for very different organisms, even though the earlier embryonic stages may have been dramatically different. This led Strähle to speculate that the presence of highly conserved upstream and intronic sequences, often considered a hallmark of conserved enhancers, may actually have nothing to do with the binding of transcription factors.

Moving from eye induction to eye differentiation, Bill Harris used four-dimensional microscopy to follow clones of retinal cells as they arise in the retina. This approach uses reporters that cause different retinal cell types to express different colored fluorescent proteins. In one experiment, individual *atoh7*:*gfp *progenitors were transplanted into an unlabelled retina. The resulting clones nearly invariably comprised one ganglion cell and one cell of an undetermined type. This suggested that *atoh7*-expressing progenitors may generate an intrinsically fixed lineage. To challenge this point, the same cells were transplanted into a mutant retina where the population of ganglion cells is heavily depleted. In this situation, many of the transplanted cells generated two ganglion cells, suggesting that the decision to form a ganglion cell or another cell type is not intrinsic to the precursor cells but is influenced by the environment, that is, by the relative numbers of cell types already present.

Harris speculated that the number and arrangement of cells that differentiate as specific types of retinal neurons, such as ganglion cells, may be determined by the number of other neurons that 'compete' with them and 'feed' them, much like the number and spacing of predators in the wild depends on competition for a finite prey population. This type of selection mechanism could ensure that the appropriate numbers of photoreceptors, bipolar and ganglion cells are always present, and may provide insight into how the relative proportions of neuronal cell types evolve to specific requirements (for example, diurnal versus nocturnal style of life).

Felix Loosli expanded on eye field specification and subsequent morphogenesis. The eye fields are defined at neural plate stages as parts of the diencephalon that will evaginate to form the optic cups. Loosli examined the roles of the transcription factor encoded by *rx3 *in the formation of the eye fields in medaka and in zebrafish. This gene is expressed very early in the presumptive fields, and mutations in both species result in an eyeless phenotype.

The generation of an *rx3*:*gfp *transgenic line allowed the examination of cell movements during the transition from neural plate to optic cup. Surprisingly, the formation of the optic cup is not a simple evagination process. Eye precursor cells behave differently from other central nervous system neurons as soon as the convergence of the neural plate, remaining stationary as they are passed by their neighbors and predetermining the site of evagination. At a later stage eye precursors actively migrate away from their region of origin to form the optic cup. Evagination can thus be viewed as tissue morphogenesis driven by the active behavior of individual cells, rather than as a supracellular process globally determined by external constraints.

Kristen Kwan took over the session to discuss the transition between the early evagination process and the fully formed optic cup. Using fluorescent membrane and chromatin markers, she described eye morphogenesis in live embryos: soon after the appearance of the wing-like evagination of the lateral forebrain, the optic stalk constricts, generating a 'flipper'-like structure. The lateral part of the optic vesicle will become neural retina, while the medial aspect will form pigmented epithelium. Next, the lens is induced by the neural retina, initiating a complicated torsional movement whereby the retina enwraps the freshly induced lens [3]. Photoactivated Kaede protein allows analysis of cell behavior at the single cell level [4]. Cells in the presumptive neural retina appear to actively migrate for a significant period before taking on a stereotyped, columnar epithelial morphology by the end of optic cup formation.

Her description of early eye morphogenesis suggests that this process involves a very complex sequence of morphogenetic events not unlike the transformation of insect imaginal discs into adult parts such as thorax and wing. To date, such wide-scale changes in tissue shape are not understood in any system, and even the relatively simple process of germ band elongation in fly embryos is still poorly understood. Sophisticated imaging tools may make eye morphogenesis amenable to genetic dissection, and Kwan demonstrated how time-lapse analyses using different fluorescent reporters will facilitate this analysis.

## Sensory cell differentiation

Sensory cells develop a transducing system that allows them to transform physical stimuli (light, mechanical displacement) into electrical input. This places special constraints on their differentiation program: they must organize synapses that are capable of sustained activity, they have to control the size of their transducing apparatus to provide a reliable estimate of stimulus intensity, and they must have the capability to optimally occupy body space to provide complete but non-redundant information.

Monte Westerfield concentrated on a particular set of genes that cause degenerative deafness as well as blindness, the Usher genes. In humans, mutations in these genes produce deafness, as well as photoreceptor degeneration. Westerfield showed that inactivating the fish homolog of any of the known human Usher genes also leads to functional blindness, making the fish an appropriate model to better understand the basis for the human disease. This result led him to develop the idea that degenerative diseases may reveal developmental mechanisms that have gone awry.

The Usher proteins all contain PDZ domains and/or PDZ binding domains, suggesting that they probably interact to form a large protein complex involved both in hearing and in vision. One possible role of the Usher proteins may be to assemble the ribbon synapses that are exclusively assembled in photoreceptors (and bipolar cells) in the retina and in hair cells in the inner ear. Ribbon synapses are a specialized type of glutamate synapse that are suitable to handle graded signaling over long times. Impairment in the assembly or maintenance of these specialized synapses may ultimately lead to cell damage and loss, thus explaining the defects associated with the Usher syndrome.

Jarema Malicki focused on another common feature of photoreceptors and hair cells: the presence of apical cilia. One of the most striking aspects of ciliated receptors is their complex polarized organization. Malicki has discovered a class of mutations that produce a loss of surface area in either the ciliary or non-ciliary compartment of the apical surface. Molecular analysis of these mutations has identified members of the Crumbs complex, implicated in defining apical domains in *Drosophila *and mutated in human retinal disease. Ongoing characterization of these genes is revealing new regulators of this important process.

Another crucial aspect of ciliated sensory cells is the architecture of the cilium. Some sensory neurons feature a structurally simple cilium while others, such as vertebrate photoreceptors, for example, differentiate complex membrane folds on their surface. Cilium differentiation relies on a specialized transport system that transfers material to and from the ciliary compartment (for example, many millions of rhodopsin molecules are transported daily into the ciliary compartment in the case of photoreceptor cells). Transported molecules move as membrane-linked densities, and transport itself depends on a set of so-called intraflagellar transport (IFT) proteins.

Numerous mutations in zebrafish, including some in IFT genes, have been shown to disrupt cilium maintenance. Their analysis reveals that genetic mechanisms of ciliogenesis are rather diverse and that even structurally simple cilia are not all equal when it comes to the genetic regulation of their formation. In recent years, the list of cilia functions has been rapidly expanding. In keeping with this trend, the analysis of zebrafish mutants suggests that photoreceptor cilia, in addition to their well-established role in sensing light, may also be involved in the regulation of photoreceptor gene transcription – perhaps to adjust the amount of protein synthesis to environmental conditions.

Alvaro Sagasti re-examined the basic problem of 'tiling': how are two-dimensional fields compartmentalized into subsets of abutting sub-fields? He used as a model system the innervation of the head skin by trigeminal neurons. Trigeminal neurons extend neurites, usually called axons even though they are not presynaptic, which extend and ramify until the head epidermis is entirely innervated. He showed that repulsive interactions between left and right neurons mediate the almost perfect ipsilateral confinement of expanding axons [5], and are also involved in setting up the topology within the trigeminal ganglion

**Figure 3:**
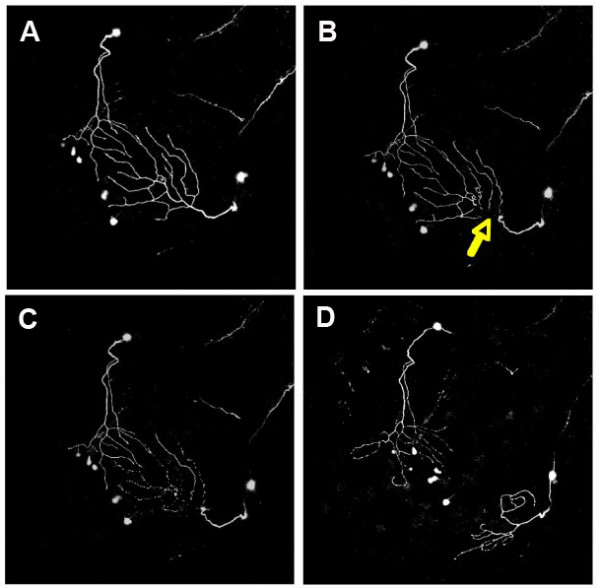
Imaging peripheral arbor re-innervation *in vivo*. Confocal projections showing dorsal views of two trigeminal axons, visualized with GFP, in a 54 hpf zebrafish embryo at several time points during a two-photon axotomy experiment. Anterior is to the bottom left. Images are each 420 microns across. **(a) **Twenty minutes before axotomy. **(b) **Approximately 20 minutes after axotomy. Yellow arrow points to site of axotomy. **(c) **Two hours after axotomy, the distal portion degenerates. **(d) **Robust regrowth is apparent 12 hours after axotomy. Figure provided by A Sagasti.

Taking advantage of the accessibility of the trigeminal neuritic trees, he then examined what happens after neurite severing. The availability of fluorescent reporter lines makes it possible to follow the process of reinnervation in real time. If the ablation is made at a time when the tiling is still being set up, both the amputated neuron and its neighbors extend into the emptied region and contribute to its innervation, resulting in an expanded field for the neighbors and a shrunken field for the amputated neuron. The real surprise came, however, from later ablations. If a branch is cut after tiling has been fully established, all surrounding neurites react to the change but somehow they are unable to invade the emptied region (Figure 3). Movies clearly reveal the reluctance of the exploring tips of neighboring neurites, as if this were haunted territory [5]. Whether this reluctance to invade the deprived region is due to some inflammatory response related to the scavenging of neuritic debris by macrophages, or to remnants of the repulsive substance, or to some yet unknown factor, is an open question.

## Collective cell migration

A recurrent theme of the meeting was the role and mechanism of cell migration, a process that plays a crucial role in the development of the cerebellum (R Köster), of the hindbrain (H Okamoto and M Rossel), of the eye (K Kwan and F Loosli) and of the lateral line (T Piotrowski, D Gilmour and C Dambly-Chaudière). The buzz expression 'chain migration' was often used to emphasize the difference between these collective processes and individual cell migration, although intense discussions demonstrated that an operational definition of 'chain migration' has yet to be given.

Tatjana Piotrowski set the stage for the following talks by providing an excellent and lavishly illustrated introduction to the lateral line system, its function and its development. She concentrated on the most intriguing part of this development, the long-range migration that gives rise to the posterior lateral-line system (PLL). In this process, a primordium arises adjacent to the otic vesicle, moves to the tip of the tail and deposits in its wake five to six small groups of cells, each of which will form a neuromast.

Piotrowski provided evidence that Wnt signaling is active in the migrating PLL primordium, and that a mutation affecting Apc, a key regulator of Wnt signaling, results in the deposition of a disorganized stream of cells instead of the precise pattern of neuromasts. The human homolog of the affected gene has been implicated in colorectal cancer, adding one more to the ever-expanding catalog of cancer-associated genes that are also involved in primordium migration.

The Apc mutant shows no obvious defect in cell differentiation nor in the pattern of expression of other genes already known to play important roles in primordium migration, suggesting that Apc may be more directly involved in the organization of the primordium than in migration *per se*. Even though Apc plays roles in other processes besides Wnt signaling, work in the Piotrowski lab has demonstrated that the lateral line phenotype in *apc *mutant embryos is exclusively caused by Apc's function in regulating Wnt signaling.

Darren Gilmour introduced his talk with breathtaking movies that illustrate how local interaction can mediate large-scale coherent movements: he showed how a school of thousands of fish fans out from an intruding diver or how a large flock of birds reacts instantly to the arrival of a prey bird as if the entire school and flock were a single organism, even though as Gilmour noted very few of the fish and birds actually saw the arriving danger.

Gilmour focused on the PLL primordium as a typical example of collective cell migration. Using a GFP reporter line specific for lateral line cells (Additional file 1), he transplanted wild-type cells into mutant embryos where the primordium cannot migrate, due to a mutation in the chemokine receptor CXCR4 (which drives migration in response to a stripe of its ligand, SDF1). He observed that a small number of CXCR4-positive cells, or even a single one, is sufficient to drive migration of the entire primordium. He concluded that the primordium is intrinsically polarized, and that the presence of CXCR4-positive cells at one edge is sufficient to organize the entire group of cells.

Gilmour also showed that the CXCR4-negative cells are not simply pulled by the leading cells, but actively contribute to the migration. Experiments using a laser to cut through the primordium confirmed that both the leading and trailing cells play a role in migration, as migration of either group stopped as long as they remained separate but resumed whenever both parts were reunited. Gilmour described the expression of another receptor of the chemokine SDF1, CXCR7. Contrary to *cxcr4*, the gene *cxcr7 *is expressed specifically in the trailing edge of the primordium. Its inactivation is also detrimental for migration, much as the inactivation of *cxcr4 *(Additional files 2 and 3). One possible explanation for the phenotypes is that CXCR4 is controlling the pulling of the primordium from the front, while CXCR7 would regulate a push from the back.

**Figure 4:**
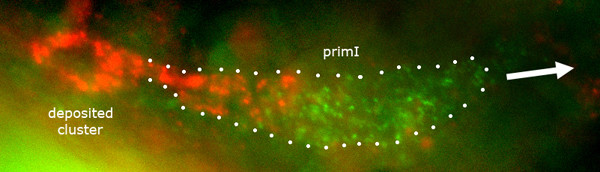
Expression of *cxcr4 *and *cxcr7 *in the migrating primordium. Double *in situ *hybridization with a *cxcr4b *probe (green) and a *cxcr7 *probe (red). *cxcr7 *is exclusively expressed by cells in the trailing region and in cells that are being deposited, while *cxcr4*b is more ubiquitously expressed (albeit at a higher level by the cells in the leading region). White dots outline the migrating primordium, primI. The direction of migration is shown by the white arrow. Picture provided by C Dambly-Chaudière and M Rossel.

Christine Dambly-Chaudière reported similar observations on the expression of *cxcr7 *(Figure 4) and on its loss-of-function phenotype. She also examined the interactions between the two receptors and found that in the absence of CXCR4, the expression of *cxcr7 *expands into the leading region of the primordium. The largely complementary patterns of expression of *cxcr4 *and *cxcr7 *seem, therefore, to result from antagonistic interactions between the two receptors.

Based on the expression of *cxcr7 *in the trailing cells and on its repression in the leading cells, Dambly-Chaudière proposes that CXCR4 drives the migration and that CXCR7 imposes directionality to this migration by making SDF1 less available to the cells in the trailing region. Thus, CXCR7 would behave as a member of a newly recognized group of chemokine receptors known as 'atypical', which bind and sequester their ligand without producing the usual signaling cascade. Atypical receptors are thought to regulate ligand availability at a very local level and thereby to modulate the activity of 'typical' receptors. Sequence comparisons between human and fish CXCR4 and CXCR7 reveal indeed a complete divergence in the signal-mediating C-terminal end of CXCR4 and CXCR7, suggesting a major difference in signaling capability.

## Mechanosensory systems: hair cells in lateral line and ear

An essential feature of mechanosensory hair cells is their planar polarization. Each hair cell projects a single microtubule-based kinocilium and several actin-based stereocilia from its apical surface with a polar organization, with the kinocilium displaced to one side. Hernan Lopez-Schier showed that each neuromast has a plane of mirror symmetry so that all hair cells on one side of the neuromast point in one direction and those on the other side point in opposition. In zebrafish with mutations in planar cell polarity genes such as *vangl2*, this organization is disrupted.

Lopez-Schier next examined the development of this polar organization. He took advantage of the fact that polarization is restored when new hair cells differentiate after ablation by exposure to aminoglycoside antibiotics. Using GFP transgenics, he demonstrated that pairs of hair cells are formed from symmetrically dividing precursors. The precursors divide along the axis of polarity and their progeny end up with their stereocilia pointing in opposite directions. Interestingly, the polarity of precursor cell division is not obviously disturbed in *vangl2 *mutants, even though hair cell polarity is randomized, suggesting that several mechanisms contribute to the generation and maintenance of polarity.

Miguel Allende continued on the theme of hair cell regeneration, discussing the recovery of lateral line hair cells after exposure to copper. Copper mining in Chile results in high amounts of copper ions in the water, and its effects on wildlife (and humans) is of concern. Allende demonstrated dose-dependent effects on neuromasts after copper exposure, with hair cells lost at lower doses and additional loss of support cells at higher doses.

**Figure 5:**
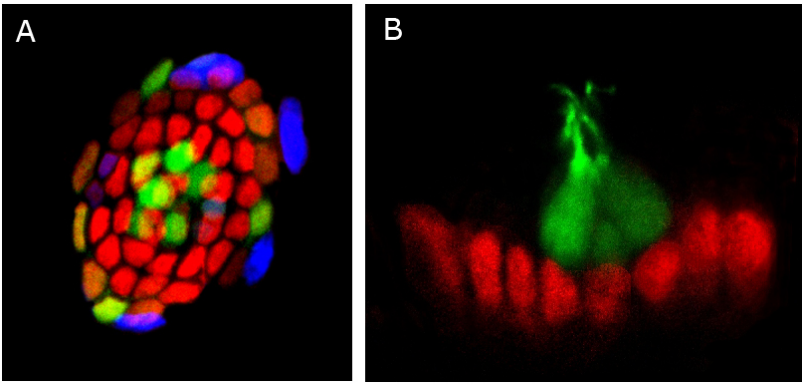
Distribution of Sox2 in lateral line neuromasts. **(a) **Confocal image of a zebrafish lateral line neuromast. Mantle cells and hair cells are labeled by GFP (green) in this transgenic line of zebrafish. Fluorescent immunostaining reveals expression of the neural progenitor marker protein Sox2 (red) and BrdU incorporation (blue). Cell division is occurring mostly in the periphery of the neuromast. **(b) **Confocal image of a zebrafish lateral line neuromast inmunostained to detect the neural progenitor marker protein Sox2 (red) and GFP (green) in hair cells. Note that these two markers do not overlap, suggesting that Sox2 is not present in differentiated cell types in neuromasts. Pictures provided by M Allende.

Allende provided evidence that hair cells are rapidly replaced after copper exposure, using GFP transgenic lines. Hair cells are restored without proliferation after exposure to lower doses of copper. In contrast, extensive proliferation precedes regeneration after exposure to higher copper levels. These observations led Allende and colleagues to examine the expression of markers expressed in neural stem cells. Allende reported that *Sox2*, a gene encoding a transcription factor expressed early in neural tissue and involved in stem cell maintenance, is expressed in support cells within the neuromast (Figure 5).

**Figure 6:**
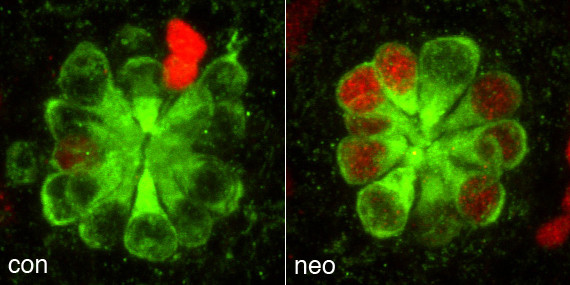
Control (con) and neomycin (neo) treated zebrafish lateral line neuromasts 72 hours after antibiotic treatment. Regenerated hair cells stained with anti-myosin VI antibody (green) are derived from proliferative precursors that incorporated BrdU (red). Figure provided by D Raible.

David Raible presented work from his lab on the regulation of hair cell regeneration. Killing hair cells with aminoglycoside antibiotics results in hair cell replacement, the vast majority derived from proliferating precursors (Figure 6). Hair cell loss triggers a wave of proliferation that subsides before functional differentiation. Raible suggested that more than one feedback mechanism may be in place, from mature hair cells that suppresses precursor proliferation and from immature replacement cells that restores quiescence.

Raible presented results from experiments exploring whether Notch signaling plays a role in precursor inhibition. The Notch signaling pathway is the key element of a lateral inhibition mechanism involved in many aspects of development, and has a well-described role in regulating the number of hair cells during the initial formation of the lateral line. Altering Notch signaling has no effect on functionally mature neuromasts, but dramatically altered hair cell number after damage. These results suggest that a recapitulation of the initial developmental program is involved in regeneration. As newly generated hair cell precursors express the Notch ligand Delta, they suppress continued proliferation of precursors responding to the initial hair cell loss.

The talks in this session all dealt with superficial neuromasts and provided complementary observations allowing an emerging picture of the continuous process of hair cell generation and polarization within the neuromast. New hair cells integrate into the existing structure with proper polarity, in part by symmetrical division across the neuromast midline. Newly generated hair cells halt further production of hair cells by activating Notch signals. There may be several populations of accessory cells that differentially express stem cell markers such as Sox2, and have so far been lumped under the category of 'support' cells.

Jacqueline Webb dealt with the formation of another type of neuromast, the so-called 'canal neuromasts'. A subset of superficial neuromasts on the head become embedded in canals at metamorphosis, the transition between larval and juvenile forms that takes place when the fish is about one month old (11–12 mm long). Contrary to surface neuromasts, which are sensitive to water velocity, canal neuromasts are most sensitive to acceleration. This dual perception of velocity and acceleration may allow the lateral line system to discriminate between different types of stimuli.

Canal neuromasts are present on the head but not on the trunk and tail of adult zebrafish (which is the reason why zebrafish are assigned by ichthyologists to the small category of fish that have no lateral line). Head canal neuromasts expand greatly during juvenile growth and become very elongate, as is usually the case for canal neuromasts in other fish species. Zebrafish canal neuromasts are unique, however, in that their long axis is orthogonal to the canal axis (much as the cristae of semicircular canals), while in the species examined to date the long axis of the neuromasts parallels the axis of the canal. The reason underlying this difference is anyone's guess, but the finding illustrates how adaptive interpretations based on a limited sampling of species may be misleading.

The continuous growth of the fish requires that bones and canals, which are embedded in those bones, are remodeled throughout life. Webb examines this process by focusing on the two main players: osteoblasts that form new dermal bone, and osteoclasts that allow bone resorption. She finds that osteoclasts are present on the inner surface of the head canals, opening the way to a genetic analysis of how this remodeling is controlled.

Tanya Whitfield presented work from her group on development and evolution of the inner ear. Contrary to the mammalian inner ear, the zebrafish otic vesicle does not form by invagination of an epithelial placode but by cavitation, whereby a mesenchymal mass of cells becomes organized as an epithelium surrounding a central lumen. Whitfield showed that correct patterning of the A/P axis of the otic vesicle depends on Hh signaling, as the ear shows a duplication of anterior elements in embryos in which Hh signaling is down-regulated, and a duplication of posterior structures when Hh signaling is elevated in the embryo.

These duplicated phenotypes are reminiscent of the relatively symmetrical inner ear found in the lamprey. The similarity is only superficial, however; analysis of molecular markers and hair cell polarity patterns in the lamprey demonstrates that the otic vesicle in this species actually has significant asymmetries about the A/P axis, and closely resembles the developing zebrafish ear. The two separate sensory patches present in the zebrafish otic vesicle would, therefore, result from the splitting of a single sensory patch present in the common ancestor of lampreys and teleosts, rather than from the development of a new patch. Splitting of an ancestral patch appears to have been dependent on the acquisition of a new domain of *otx1 *expression in the ear: the two sensory patches are juxtaposed in zebrafish embryos in which Otx1 function has been knocked down by morpholino injection.

How Hh signaling acts to polarize the otic vesicle is not clear, since A/P polarity can be detected as soon as cavitation begins, at a stage when the long axis of the vesicle is parallel to the major sources of Hh signaling, the notochord and floorplate. Furthermore, an excess of Hh signaling is detrimental to the establishment of the other two axes (D/V and M/L) as well. It seems likely, therefore, that other factors act in addition to Hh proteins to establish the axes of the inner ear.

## Integrative brain

**Figure 7:**
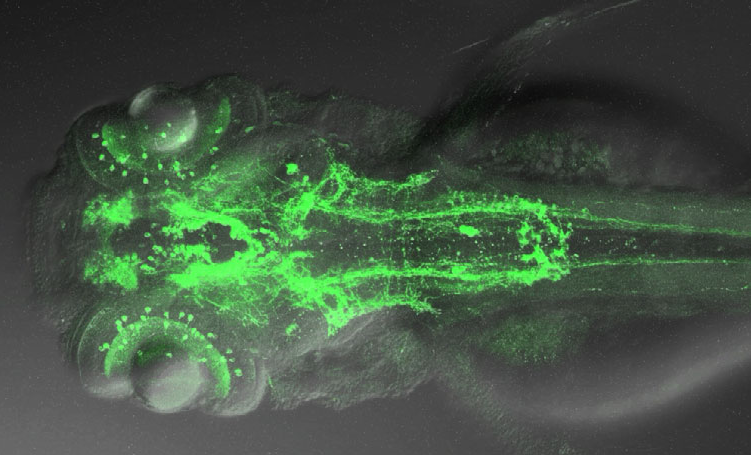
Projection on the horizontal plane of a Z-stack movie shown by J Schweitzer, illustrating a whole mount 3 dpf embryo labeled with an antibody against tyrosine hydroxylase. This picture was generated by S Ryu and W Driever.

After dealing with the development of sensory capabilities, the talks focused on the integrative capabilities of the brain. Jörn Schweitzer introduced the change in focus with a nice movie exploring the ordered complexity of the dopaminergic system and its connectivity (Figure 7). He demonstrated that Robo2 mediated signaling is required to specify lateral positioning of descending longitudinal projections in the hindbrain, which are derived from dopaminergic neurons located in the ventral diencephalon. These projections, which normally grow within the medial longitudinal fascicle, are shifted towards the midline in *robo2 *mutants. The shift does not result from a general remodeling of the hindbrain in *robo2 *mutants because other axonal trajectories such as reticulospinal projections growing in close vicinity to the dopaminergic fibers retain their normal positions.

The connectivity of dopaminergic neurons relates directly to behavior as demonstrated by the analysis of zebrafish mutants affecting the specification of dopaminergic neurons. Schweitzer showed that the *orthopedia *gene, *otpa*, is a key transcriptional regulator of ventral diencephalic dopaminergic neurons in fish. The lack of *otpa *activity leads to a strong reduction of dopaminergic diencephalic neurons and their respective projections into the spinal cord. Behavioral analysis of *otpa *mutant larvae indicates defects in the motor system, such as a delay in the initiation of movements

The sensory neurons of the PLL extend a peripheral axon to the migrating primordium (Additional files 4 and 5) and a central axon to the hindbrain (first-order projection). Post-synaptic neurons in the hindbrain then transmit PLL information to other brain centers (second-order projection). Hitoshi Okamoto studied this second-order projection with the aim of understanding the rules that direct the ordered development of a brain, including its intrinsic connectivity. He used two reporter lines to dissect the major components of the projection. One component comprises neurons expressing *gad2*, a marker of inhibitory neurons. The analysis of *gad2*:*gfp *neurons reveals that they project to the contralateral side of the hindbrain, presumably allowing for an instantaneous comparison between left and right inputs. This comparison may be important to help fish swim against water flow (and explain why river fish are not washed out in the oceans).

A second component is revealed by the expression of the *zic1 *gene. A transgenic line where GFP is put under the control of the *zic1 *promotor reveals that GFP-positive neurons are born from *atoh1a*-dependant progenitors, and that they project to a structure known as the torus semicircularis, in the contralateral midbrain. This projection appears homologous to the mammalian projection from inner ear to inferior colliculus and is the starting point to all subsequent (third-order) projection to higher brain centers in adult fish. Inactivation of *atoh1a *removes this component of the projection without affecting the contralateral projection of the *gad2*+ neurons.

**Figure 8:**
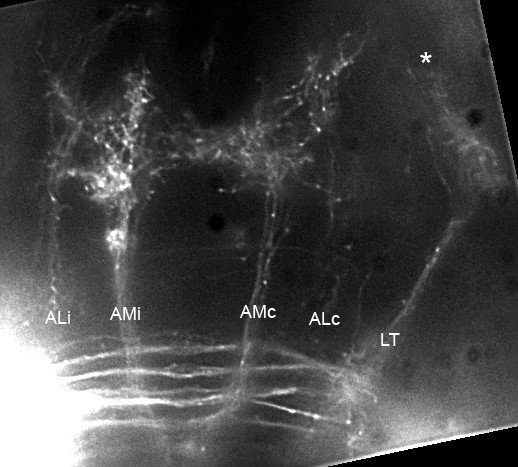
Second-order projection from the lateral line, at the level of rhombomeres 1–2. Two large branches, AMc and AMi, extend symmetrically on either side of the midline towards the oculomotor nuclei and nuclei of the Median Longitudinal Fascicle (MLF). More laterally, two ill-defined branches extend to forebrain nuclei (ALc and ALi). A fifth branch extends to the contralateral torus semicircularis (LT). At the anterior edge of the LT branch, a few fibers extend dorsally (asterisk) into the tectum. Dorsal view, composite of several focal planes. The complete Z-stack is shown as Additional file 7. Figure provided by A Ghysen.

Alain Ghysen also examined the second-order PLL projection with the slightly different aim of understanding how sensory inputs are handled by the brain and integrated in a coherent behavior. He relied on DiI labeling of the synaptic field formed by the afferent neurons that innervate the posterior lateral line. He confirmed the data of Okamoto about the early presence of a projection to the torus semicircularis in 4 dpf larvae (Additional file 6). In addition, he observed several other components projecting to midbrain and forebrain targets (Figure 8; Additional file 7). Some of these components correspond to projections from the vestibular system in amphibians or mammals. Both lateral line canals and vestibular semicircular canals act as acceleration detectors, as mentioned earlier by Jackie Webb, and may synergize in some situations to help fish to keep an accurate control of their course over long distances.

The presence of projections to targets that were not previously documented as second-order lateral-line targets in adult fish prompted Ghysen to speculate that the complex pattern found in the zebrafish embryo might represent an ancestral scaffold from which different groups of animals have selected various subsets for their own purposes. Given that neurons projecting to different targets seem to be independently specified, as shown by Okamoto, this early pattern may be individually tailored for the species during subsequent larval development.

## Brain laterality

Theresa Burt de Perera studies how blind fish manage to build a large-scale map of their surroundings. Using either plain or annular tanks and using small Lego blocks as landmarks she showed that blind *Astyanax *speed up when they encounter a new feature (Additional files 8 and 9), allowing her to decide whether or not changes or permutations of landmarks are recognized as new features. She demonstrated an amazing degree of sophistication, such as the capability to discriminate between an isosceles triangle A, B, C (clockwise) and one where B and C have been permutated (A, B, C counterclockwise), implying that the cave fish can encode the order of a landmark sequence. She hypothesized that large-scale maps could be built much as jigsaw puzzles, by linking 'local' maps (areas that are within a fish's perceptual range) together in order. This would explain why blind fish tend to swim along objects rather than in plain water – contrary to naive intuition.

Among other interesting observations, Burt de Perera showed that blind fish expose preferentially their right side to new stimuli, in a striking parallel to the right eye preference showed by normal fish, pointing to a left hemisphere dominance for exploratory behavior. There is, however a small proportion of the population that consistently prefers its left side (the 'left-finned' fish). An interesting question (S Wilson) was whether the proportion is the same in blind and eyed forms – because the blind form is less social than the eyed form, a difference in proportion might point to an effect of socialization in setting the balance between left-finned and right-finned fish

A similar left-right bias was also noticed by Anukampa Barth (poster session) when she examined the balance between fear and curiosity of fish presented with an unexpected stimulus on either the right or the left eye. It will be interesting to find out whether these 'psychological' differences between right and left brain are associated with lateralization of the habenular system discussed by Wilson and Okamoto.

The question of brain laterality was taken to a more developmental level by Wilson and Okamoto. Steve Wilson discussed the origin and selective advantages of laterality. As he explained, very little is presently understood about the population biology and evolution of laterality. The most basic questions, such as whether bilateral symmetry is a default state or whether it is a feature superimposed on a fundamentally asymmetric structure, still remain quite open. Even more importantly, while it is clear that asymmetries pervade the organization and function of brain and body alike, what exactly is their adaptive value (if any) is still a matter of speculation – an ingredient that fortunately was abundant throughout the meeting.

Wilson concentrated on the special case of a forebrain structure, the habenula, which provides input to a midbrain structure, the interpeduncular nucleus (IPN). The axons from the left and right habenulae innervate two non-overlapping regions of the IPN [6]. This asymmetry is related to the presence, on the left but not on the right side, of the parapineal nucleus that is formed by out-migrating pineal neurons and projects exclusively ipsilaterally, to the left habenula.

Genetic analysis suggests that the lateralization of the habenula comprises several components. A first step would be the lateral migration of pineal neurons to form the parapineal nucleus, a step that appears to depend on the bilateral expression of *fgf8*. A second step is the choice of whether the parapineal will be localized left or right. This step requires *nodal *function. In the absence of nodal, however, the migrating cells decide to migrate either right or left, instead of forming two small parapineal nuclei on either side of the midline. Thus, there must be at least one additional step forcing the cells to migrate to only one side of the brain even in the absence of nodal, setting up this fundamental asymmetry of the brain.

Hitoshi Okamoto showed that the difference in connectivity between left and right habenula depends on a large size difference between two subnuclei, a medial one that is prominent in the right habenula and projects to the ventral IPN, and a lateral one that is prominent in the left habenula and projects to the dorsal IPN. He then examined the pattern of neurogenesis of the habenula precursor cells by using both bromodeoxyuridine (BrdU)-pulse labeling and cell-lineage tracing using photoconversion of differentiated neurons in a HuC-Kaede transgenic line. He showed that neurogenesis follows different time courses in the prospective left and right habenular regions, suggesting that lateralization is set up very early in brain development.

In order to determine whether this temporal difference in neurogenesis is responsible for the differences in neuronal identity between medial and lateral subnuclei, Okamoto used heat-shock activation of Notch to block neurogenesis at different times. He observed that a marker for the early neurons forming the lateral subnucleus (prevalent on the left side) is markedly depleted when Notch activation is forced early on, while a marker for the late neurons that form the medial subnucleus is ectopically expressed on both sides of the midline, instead of being largely restricted to the right side. The link between the asymmetric localization of the parapineal discussed by Wilson, and the asymmetric neurogenesis reported by Okamoto, remains to be discovered.

Okamoto also examined the tectobulbar tract that connects the optic tectum to the reticulospinal neurons in the hindbrain using another transgenic line, Tg [brn3a:GFP]. Because reticulospinal neurons in turn control spinal motoneurons, this pathway is an essential component of the visual control of motion. The neural circuits controlling visuo-motor transformation might be lateralized as suggested by the observation that when fish approach a new object they do so very carefully and use their right eye (much as in blind cavefish using the right lateral line, as mentioned above). On the contrary, they display no ocular dominance when they encounter a well-known object. No morphological asymmetry could be detected in this pathway, however, leaving open the possibility that, in this case, lateralization is at the level of function. The possibility of labeling single tectal efferent neurons (with a Cre-lox system; Additional file 10) may help understand how this tectobulbar projection is organized.

## Behavioral brain

Suresh Jesuthasan examined fish's fear and argued that the concept of fear and the effect it triggers is probably not very different in fish and in mammals. Indeed, his movies of frightened fish were immediately 'understood' by the audience – the increase in swimming speed, frantic avoidance of the frightening stimulus and desperate attempts to hide in some bottom crevice are all very eloquent. This behavior turns out to be induced by the release of a 'fearomone' – a pheromone present in the skin such that if one fish is bitten all its fellows are quickly warned of the lurking danger. Jesuthasan described how this behavioral assay could be used to screen for mutations that alter response to this alarm substance ('Schreckstoff') present in the skin of all ostariophysians fish.

Jesuthasan described electroporation techniques developed in his lab to label and follow cells within the nervous system. These electroporation methods are likely to be useful to a number of labs interested in dissecting circuit function. More generally, this talk made it clear that new techniques for the analysis of brain circuits will be critical to understanding complex individual and social behaviors.

**Figure 9:**
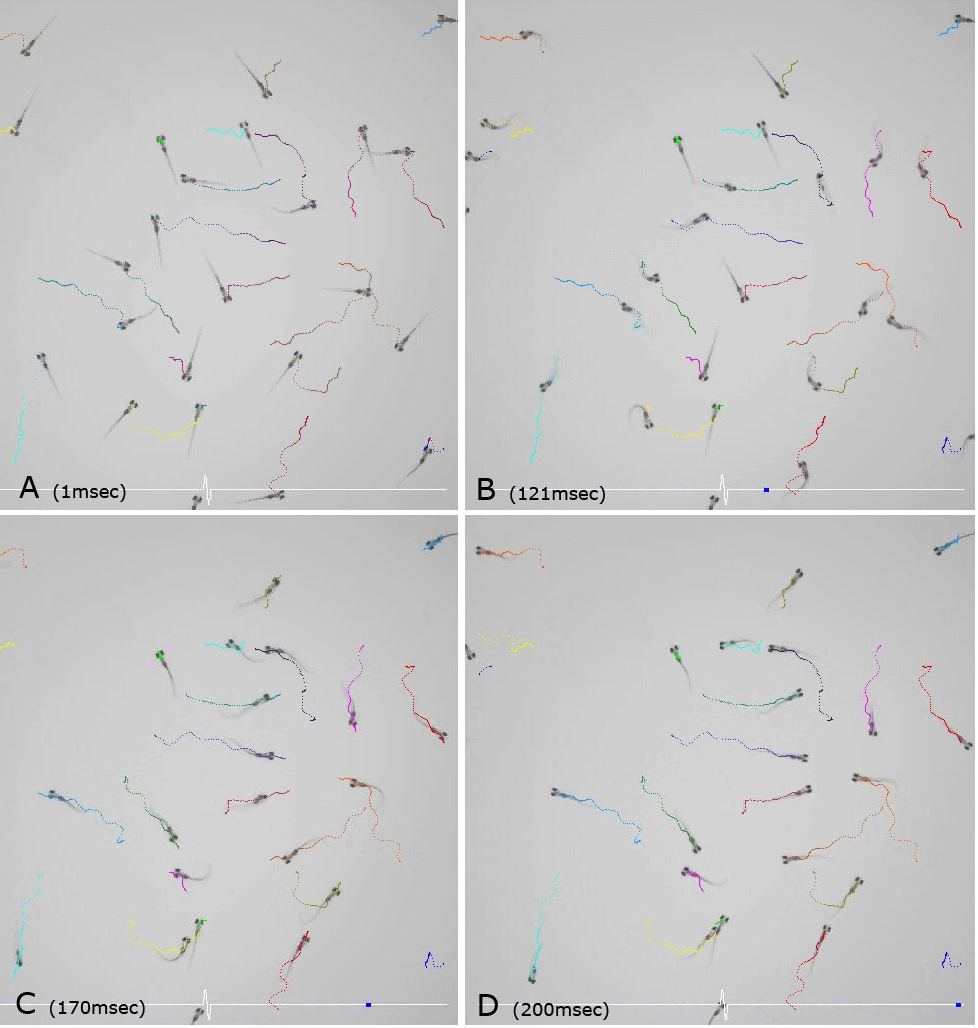
Four still pictures at different stages of the startle response, taken from Additional file 11. The pictures were taken at **(a) **1, **(b) **121, **(c) **170 and **(a) **200 ms after stimulation. Figure provided by M Granato.

Michael Granato took on the same theme in embryos – where the manifestation of fear is called 'startle response'. The startle response is induced by a sudden, intense sensory stimulation and comprises a very stereotyped set of muscle contractions called 'C-turn' (Figure 9 and Additional file 11), the result of which is that the embryo flees away from the stimulus. This response is extremely rapid (latency about 2–4 ms) and is mediated by a giant reticulo-spinal neuron, the Mauthner cell.

Granato's question was, can this innate strong response be modulated? It is known in humans (and very convincingly demonstrated by Granato through movies exploring the startled behavior of unsuspecting graduate students) that sensory information can be 'gated', that is, that previous experience can modify our response to a frightening stimulus. This 'pre-pulse inhibition' is reduced in human schizophrenia, Tourette and a mild version of autism, Asperger's syndrome.

The conclusion of the work is that a non-frightening pre-pulse can inhibit startle response by as much as 70% as long as it is provided less than 100 ms before the startling stimulus. Similar to the situation in humans, this pre-pulse inhibition is enhanced by agents such as nicotine and haloperidol, which are known in humans to reduce fright. Granato described his ongoing search for mutations that alter the startle response and may yield useful cues about the human response and its modulation.

Florian Engert explored another aspect of cross-modality: how exactly does visual information impinge upon orientation to (and swallowing of) prey? He showed that visual control of movement is already very sophisticated in the early larva. For example, a spot corresponding to a visual angle of 1 (about the apparent size of a paramecium) elicits a tracking behavior, a spot of 5 elicits an escape behavior, while a spot of 20 elicits an optomotor response. These differences appear innate, implying a high level of genetic control of visual processing circuits.

Detailed analysis of prey-tracking also revealed interesting subtleties. For example, tracking of the prey by rotating an eye is achieved by smooth continuous movement of the eye, while tracking by rotating the body axis is achieved in jerks. Some aspects of this tracking are rather puzzling: for example, why is it that the movement of an eye tracking a moving paramecium is accompanied by the antagonistic (apparently meaningless) movement of the other eye? Clearly we need to understand more of the underlying connectivity to make sense of such nonsense. In order to better analyze prey-tracking responses like these, which likely involve the coordinated action of a number of neurons, Engert is developing a system of live imaging of neuronal activity. Contrary to the usual systems based on magnetic resonance or positron emission, however, the system he is developing can allow single-cell reconstruction of neuronal activity.

This last talk reinforced the take-home lesson of the entire meeting; that methods to visualize neural development and function in real time and at the level of the single cell will probably be crucial in our attempts to understand the brain. The advent of such imaging methods in the zebrafish embryo may well represent a unique opportunity to break the genetic code of behavior.

## Competing interests

The author(s) declare that they have no competing interests.

## Supplementary Material

Additional file 1 Migration of the posterior lateral line primordium as visualized in a claudin-GFP line. Movie provided by D Gilmour Click here for file

Additional file 2 Migration of the posterior lateral line primordium in a *cxcr7 *morphant embryo. Movie provided by D Gilmour Click here for file

Additional file 3 Migration of the posterior lateral line primordium in a *cxcr4b *mutant embryo. Movie provided by D Gilmour Click here for file

Additional file 4 Time-lapse observation of a growing lateral line axon in a 28 hph embryo injected with the HuC-Kaede plasmid. Time-lapse observation of a growing lateral line axon in a 28hph embryo injected with the HuC-Kaede plasmid, as described in [7]. In this case, Kaede was not photoconverted. Movie by Hideomi Tanaka, Tomomi Sato and Hitoshi Okamoto. Click here for file

Additional file 5 Time-lapse observation of the same lateral line axon displayed in three dimensions. Movie by Hideomi Tanaka, Tomomi Sato and Hitoshi Okamoto Click here for file

Additional file 6 Second-order projection from the lateral line to the torus semicircularis. A series of 22 frontal planes, from posterior to anterior, taken from a 300 micron thick frontal vibratome section. The fibers enter as bundles (first frames) and arborize as they reach their targets. The LT branch arborizes along the torus semicircularis; as this branch reaches its most anterior extent (last frames) a few fibers escape and climb dorsally into the overlying tectum (yellow arrows). Also present in this figure is the PM branch that arborizes just anterior to the oculomotor nucleus. Movie provided by A Ghysen. Click here for file

Additional file 7 Three-dimensional stack documenting the second-order projection illustrated Figure 8, from the most ventral to the most dorsal plane (13 planes, whole-mount preparation). The dorsal planes reveal a few fibers climbing dorsally from the anterior tip of the LT branch into the deep layer of the optic tectum (yellow arrows). Movie provided by A Ghysen. Click here for file

Additional file 8 A blind Mexican cave fish leisurely swimming in an annular arena with Lego landmarks to which the fish is accustomed. This clip shows part of the last training trial (control) after the fish had been repeatedly presented with the experimental arena. Movie provided by T Burt de Perera. Click here for file

Additional file 9 The same cavefish in the same arena, after two of the landmarks had been switched (test trial), changing their order. Note how much faster the fish is swimming in the test trial than in the control, indicating that it had recognized a change in its environment. Movie provided by T Burt de Perera. Click here for file

Additional file 10 Individual efferent neuron in the optic tectum, stochastically labeled with the Cre-loxP/Gal4-UAS system. Individual efferent neuron in the optic tectum, stochastically labeled with the Cre-loxP/Gal4-UAS system described in [8]. Movie by Tomomi Sato and Hitoshi Okamoto. Click here for file

Additional file 11 The fish startle response. The time of mechanical stimulation is shown on the lower line. Movie provided by Michael Granato. Click here for file
